# Spirometry as a motivator for smoking cessation among patients attending the smoking cessation clinic of Monastir

**DOI:** 10.1186/s12889-022-13583-1

**Published:** 2022-06-10

**Authors:** Manel Ben Fredj, Behaeddin Garrach, Cyrine Bennasrallah, Asma Migaou, Hela Abroug, Wafa Dhouib, Imen Zemni, Meriem Kacem, Ines Bouanene, Asma Belguith Sriha

**Affiliations:** 1 Department of Epidemiology and Preventive Medicine, University Hospital Fattouma Bourguiba, Monastir, Tunisia; 2 Faculty of Medicine of Monastir, University of Monastir, Monastir, Tunisia; 3 Research Laboratory “Technology and Medical Imaging”, Monastir, Tunisia; 4 Department of Pneumology, University Hospital Fattouma Bourguiba, Monastir, Tunisia

**Keywords:** Smoking Cessation, Motivation, Spirometry, Tunisia

## Abstract

**Background:**

The choice of spirometry, a biomarker of lung health, as a motivator for smoking cessation is based on its fidelity in emphasizing tobacco adverse effects. Yet, there is a paucity of evidence on its efficacy, and the findings are currently inconclusive. The aim of this study was to determine whether a spirometry and lung age communication has an effect on smoking cessation rates.

**Methodology:**

We conducted a randomized controlled trial among patients who attended the smoking cessation clinic (SCC) at Fattouma Bourguiba University Hospital in Monastir, from June 2017 to February 2020. Participants were assigned into two groups, a control arm receiving standard program and intervention arm receiving a spirometry and lung age announcement along with usual care. The primary outcomes were the smoking cessation rates after one year of follow-up between the intervention arm and the control arm.

**Results:**

At one-year endpoint, a total of 456 were reachable for assessment, 236 in control group and 220 in spirometry group, which leads to a loss rate equal to 8.8%. One-year smoking cessation rate was higher among the intervention group than among control group (25.5% versus 16.5%), with a considerable statistical significance (*p* = 0.019). Lung age was significantly higher at paired comparison with chronological age.

**Conclusion:**

Smoking cessation is still a challenging procedure with a high risk of relapse, making very valuable any approach that may increase motivation in both unmotivated and motivated smokers. This study is an additional evidence for spirometry and lung age announcement as motivators for smoking cessation.

**Trial registration:**

Pan African Clinical Trial Registry database (PACTR202110595729653), 06/10/ 2021.

## Background

During the past two decades, tobacco control efforts have been proven to be successful, global tobacco use prevalence has fallen [1]. Therefore, a clear decrease of its harmful effects such as lung cancer has been noted, particularly in developed countries.

Nevertheless, tobacco use remains a major public health problem especially in developing countries and emerging economies. WHO reported more than 8 million deaths due to cigarette smoking during 2019. In accordance with global trends, prevalence of tobacco consumption among Tunisian population aged 15 has been decreased from 1997 when smoking prevalence was 30.4%, compared to 25.1% in 2016 [2]. Although this downward trend is unlikely according to experts, with a strong hypothesis of under-reporting among women and teenagers. Tobacco is still killing about 10,000 Tunisians each year [3].

Meanwhile, health structures countermeasure-procedures prove to be unsatisfying. Therefore, many innovative strategies have been designed and put into test to enhance patient motivation toward tobacco cessation. Motivation is where the biggest challenge of smoking cessation prevails, therefore medical institutions and teams are constantly developing tools to enhance patient motivation toward cessation mainly through a presentation of the adverse effects of smoking versus the benefits if quitting and assistance during the quitting procedure [4]. Many tools can be involved in this approach«smoking cessation advice and motivational support», family assisted approaches, complimentary screening, and the development of mobile applications [5].

Lung health monitoring is an objective way to explicitly show smoking adverse effect on health status. By emphasizing measurable effects of tobacco on lungs we may turning patient attention to the importance of quitting and enhance his motivation toward cessation. In fact, many interventions have been adopted to tackle this highly challenging issue, among them was pulmonary function test (PFT) or spirometry including lung age determination and communication, yet recent reviews have shown significantly controversial results [6, 7]. Among all pulmonary explorations, Pulmonary Function Tests (PFT) or spirometry was the earliest to be used as a smoking cessation motivator, a pilot study was conducted in 1978 by Rose et Hamilton where spirometry results were included in an overall score used as an indicator of a major illness or death risk [8]. In the 80s and early 90s, and with the widespread use of spirometry many other studies used spirometry to improve smoking cessation rates. Two main systemic reviews were conducted to evaluate the effectiveness of spirometry as a smoking cessation motivator, Wilt et Al., 2007 [6] and Westerdahl et Al., 2019 [7], and results were inconclusive.

This study aimed to assess the effectiveness of announcing spirometry results and lung age on smoking cessation among patients attending the smoking cessation clinic (SCC) of Monastir.

## Methods

### Study design

We conducted a randomized controlled trial (RCT), to assess the effect of adding a spirometry intervention to our standard smoking cessation program on cessation rate. Participants were assigned into two groups differing only by receiving an intervention containing pulmonary function test (PFT), a communication of its results and an announcement of the “lung age”.

### Study setting

The SCC is situated in the department of Preventive Medicine in Fattouma Bourguiba Hospital in the region of Monastir-Tunisia. This activity began on 1998.

### Study population

#### Participant selection and illegibility

Participants were smoker adults (age>=18) selected among patients who attended the SCC, from June 2017 to February 2020. We included consultants who were cigarette smokers; aged 18 and above and accepted to take part in the survey. We excluded patients with missing or incorrect contact information; patients who were unreachable after more than 3 call attempts in different occasions; loss to follow-up for any reason deceased or other; and in the intervention group, patients who were not eligible for performing spirometry test.

#### Sample size calculation

Determining the sample size needed for a two-armed design requires first the estimation of the main parameters of the outcome of this study: cessation rate and its difference detection sensibility. Previous study in the same department shows a cessation rate around 30% [9], with 5 % of risk (α) and 80 % strength in two-tailed tests, the minimum simple size at baseline should be: *n*= 500 (*n*=250 in each group). This allowed the detection of differences in smoking abstinence greater than or equal to 12 %. The sample size was estimated using BioStaTGV.

#### Randomization

Sequence of random numbers is generated by a computer. Allocation was determined by the holder of the sequence who is situated off site. Numbered sealed opaque envelopes were used for the concealment of random numbers. All patients participating in our experiment were randomly assigned to the study group and the control group.

#### Interventions

##### The standard smoking cessation program

Our SCC rotation program consists of an initiation visit and weekly monitoring during up to 6 months, if necessary, all procedures, as well as treatment, are free of charge. Patients attending SCC in Tunisia benefited from free health care (counseling and treatment programs). That measure is part of the national program to fight against tobacco use.

#### Initiation visit protocol

Via a structured questionnaire we collected demographic and biographic data, characterized smoking profile and behavior, and detailed cessation history. Personal and familial comorbidities and complains related to smoking were stated through anamnesis. A basic physical examination was performed as well, with a screening for diabetes and arterial hypertension.

All participants in both arms benefited from an educational session with detailed information about mechanisms of tobacco addiction, benefits of tobacco-cessation, potential difficulties in quitting smoking, and mode of action of nicotine replacement treatment, all with standardized speech and illustrations.

#### Pharmaceutical treatment

Clinical studies and reviews have shown that nicotine replacement products can help smokers abstain from smoking or reduce their tobacco use by decreasing withdrawal symptoms and thus can constitute an efficient smoking cessation intervention [10–13]. Therefore, it had been chosen in our clinic as main pharmaceutical intervention along with the motivational approach. In Tunisia, the unique galenic form available in the different SCC is the transdermal patch which is a sustained release form of nicotine. The therapeutic protocol, as suggested is a stepwise decrease of the dosage while starting with a maximum dosage based on previous nicotine daily intake via cigarettes; on average, one cigarette contains one mg of nicotine [14]. Then, the subsequent dosage reduction is realized by decreasing 7mg every 4 weeks.

#### Rotation procedures

The initiation visit is followed by a maintenance period with regular weekly follow-up for: Assessment of smoking abstention via CO-oximetry, up keeping motivation to stop smoking or stay abstinent, therapeutic adjustment, and detecting and treating adverse effects of nicotine patches.

##### Intervention: spirometry feedback

In addition to usual care, participants assigned to the intervention group received standardized information about their spirometry during a dedicated cession lasting approximately 30 minutes, where a spirometry was performed, a brief summary of its results and their interpretation and functional implications was given. The participant was also be informed about the “lung age” compared to the chronological age. Thus, this will illustrate the pulmonary deterioration that occurs because of tobacco use.

##### Follow-up and outcomes

Outpatient follow-up period can be extended up to 6 months if necessary, if not all patients were contacted by telephone 6 months after to determine whether they have stopped smoking, and if they have not, how many cigarettes they are smoking per day at the time and how long they have been abstinent. One year after the rotation a further phone call was made to assess once more the patient’s smoking status.

The primary outcome variable was smoking cessation 1 year after the rotation, the most suggested delay for an authentic smoking cessation [15], while the secondary outcome variables was: 6 months cessation status; the number of cigarettes/day for those who continue smoking.

### Statistical analyses

Data analysis were carried out by using SPSS (Statistical Package for the Social Sciences) version 23.0. We used mainly means and standard deviation (SD) to describe continuous variables. Qualitative variables were expressed as effective and percentages.

Comparison of continuous variables was assessed with the student’s t-test, if the data meet the assumption of normality, and otherwise with the corresponding non-parametric test. For qualitative variables comparison we have made use of Chi-squared test (χ2).

Given that the primary outcome measure is categorical in nature: smoking status after 1 year (whether patients do or do not quit smoking), the final comparison was performed using chisquared tests and was expressed as relative risk (RR). Absolute risk reduction (ARR) and number needed to treat (NNT) with the corresponding 95% confidence interval were also provided (main objective).

## Results

**Fig. 1:**
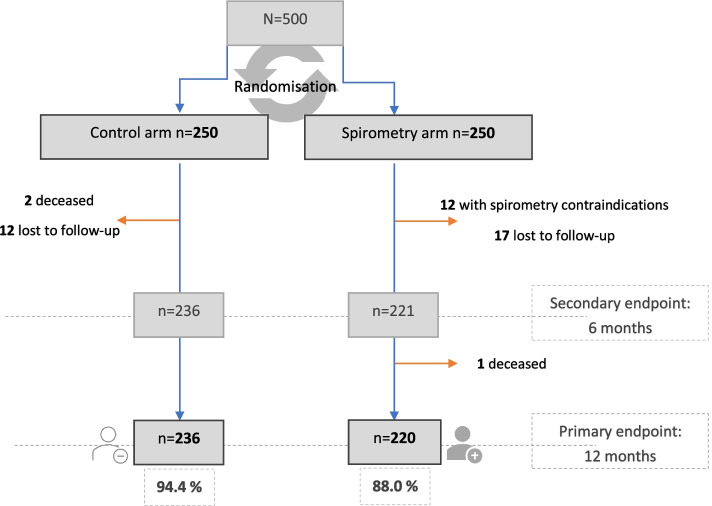
Study flow chart

A total of 500 patients were selected among attendees of Monastir’s SCC. We randomly assigned 250 participants in each group. A total of 44 were excluded by the one-year endpoint: 3 were deceased, 12 were not eligible for spirometry test and the rest were lost to follow-up. At one year endpoint a total of 456 were reachable for assessment, 236 in control group and 220 in spirometry group, that is an 8.8% of loss rate (Fig. 1).

### Baseline characteristics and between-arms comparison

#### Sociodemographic characteristics

Participants were essentially men (96%) with a mean age of 43±14.04 years old. Most of them are living within a 20 Km perimeter of the cessation clinic (91%). Patients’ educational level was quite balanced between classes, most of the patient were educated with the exception of 1% of uneducated patients. The majority of participants were active workers (67%), 8% of unemployed and a notable proportion of students (8%). When asked about having current familial and professional issues, respectively 27% and 28% had responded positively. There was no significant difference between study arms within any of the sociodemographic characteristics (Table 1).

**Table 1:** Sociodemographic characteristics

Variable	Total	Control group	Intervention group	*p*-value
Age: mean ± SD	43.65 ± 14.04	43.16 ± 15.29	44.19 ± 12.14	0.415
Sex ratio		18.08	20.36	0.836
Male: n (%)	459 (95)	235 (94.8)	224 (95.3)	
Female: n (%)	24 (5)	13 (05.2)	11 (04.7)	
Distance to clinic: n (%)				
In 20 km radios	422 (91.1)	206 (88.8)	216 (93.5)	0.101
Out 20 km radios	41 (8.9)	26 (11.2)	15 (06.5)	
Schooling level: n (%)				0.291
Unschooled	6 (1.3)	1 (0.4)	5 (02.2)	
Primary school	114 (25.1)	61 (26.4)	53 (23.8)	
Secondary school	147 (32.4)	90 (39.0)	57 (25.6)	
High school	57 (12.6)	17 (07.4)	40 (17.9)	
Two years of higher education	47 (10.4)	17 (07.4)	30 (13.5)	
Higher	83 (18.3)	45 (19.5)	38 (17.0)	
Profession: n (%)				
Active	312 (67.8)	147 (62.6)	165 (73.3)	0.136
Unemployed	39 (8.5)	27 (11.5)	12 (05.3)	
Student	39 (8.5)	26 (11.1)	13 (05.8)	
Retired	67 (14.6)	32 (13.6)	35 (15.6)	
With physical disability	3 (0.7)	3 (01.3)	0 (0.0)	
Familial issues: n (%)				
Yes	126 (27.8)	70 (30.0)	56 (25.5)	0.295
No	327 (72.2)	163 (70.0)	164 (74.5)	
Professional issues: n (%)				
Yes	120 (28)	63 (28.1)	57 (25.9)	0.669
No	324 (72)	161 (71.9)	163 (74.1)	

### Smoking profile

The mean age of cigarette smoking initiation was 17±04 years old. Regular consumption was installed at the mean age of 20±5.01 years old. Most of the clinic attendees are heavy (53 %) and super heavy (33 %) smokers, the mean cigarettes intake was 31±15 cigarettes per day with a budget of 36±15 Tunisian dinar per week (13.22±5.14 USD per week). Fagerstrom Test for Nicotine Dependency was 6.0±2.0 at mean. HAD score mean was 13.0±6.0 with the anxiety indicator being considerably higher than the depression indicator (8.4 vs 5.0) (Table 2).

**Table 2:** Smoking profile variables description and between arms comparison

Characteristics	Total	Control group	Spirometry Group	*p*-value
**First cigarette age:** mean ± SD	17.33 ± 4.71	17.11 ± 4.76	17.57 ± 4.66	0.438
**Regular smoking age:** mean ± SD	20.16 ± 4.97	19.74 ± 5.13	20.59 ± 4.92	0.463
**Cigarette/day: mean ± SD**	31.23 ± 15.88	32.13 ± 16.51	30.27 ± 15.14	0.203
Light smoker: n (%)	26 (05)	14 (05.6)	12 (05.1)	
Moderate smoker: n (%)	27 (05)	14 (05.6)	13 (05.5)	
Heavy smoker: n (%)	257 (53)	123 (49.6)	134 (57.0)	0.321
Super heavy smoker: n(%)	162 (33)	93 (37.5)	69 (29.4)	
**Pack-Year**: mean ± SD	37.07 ± 29.45	35.51 ± 31.46	38.70 ± 26.19	0.258
**Budget for smoking**: (Tunisian Dinars/week) mean ± SD	36.2 ± 14.4	37.7 ± 23.7	35.5 ± 28.2	0.378
**Fagerstrom score**: mean ± SD	6.46 ± 2.27	6.46 ± 2.28	6.46 ± 2.26	0.974
**HAD score**: mean ± SD	13.56 ± 6.71	13.82 ± 7.03	13.26 ± 6.35	0.568
Anxiety Score	8.31 ± 4.21	8.44 ± 4.28	8.17 ± 4.13	0.498
Depression Score	5.27 ± 3.59	5.42 ± 3.81	5.12 ± 3.33	0.372

It is also noted that 26.6 % of the participants live with another smoker at home and 10.76 % of them regularly use other forms of tobacco, mainly narghile (Chicha). Likewise, among the smoking behavior and lifestyle characteristics, neither subjective scales nor objective variables demonstrated significant difference between control and intervention participants (Table 3).

**Table 3:** Between arms comparison of smoking behaviors and lifestyle habits

Characteristics	Total	Control group	Spirometry Group	*p*-value
Other smokers at home: yes n (%)	135 (28.97)	76 (31.1)	59 (26.3)	0.251
Use of other forms of tobacco: yes n (%)	55 (13.78)	30 (12.3)	25 (16.2)	0.096
Coffee drink per day: mean	2.86	2.88	2.84	0.824
Meal per day: mean	2.73	2.8	2.66	0.063
Alcohol consumption: yes n (%)	137 (29.0)	77 (31.6)	60 (26.2)	0.199
Physical activity (hour/week): mean	1.01	1.09	0.91	0.106

### Clinical features

For personal medical history, 18.2 % of our patients had diabetes, 17.4 % had a cardiovascular disease, 8.1 % (*n*=39) had a pulmonary disease including 14 patients with a history of diagnosed COPD (chronic obstructive pulmonary disease) and 2.7 % had a psychiatric disorder. Nearly one third of participants had at least one chronic disease. The most frequently reported functional complains in relation with smoking were consecutively: dental damage (67.3 %), dyspnea (65.7 %), and couth (61.6 %). At physical examination, BMI was normal for 31.7 % of the participants, while over weighted and obese were 25.9% and 13.1% consecutively. High blood pressure was found in 13.9 % of the participants, capillary glycemia was found superior to 1.40 g/L in 9.5 % and superior to 1.80 g/L in 5.4 % of the cases. CO-Oximetry was 11.07 at mean. Univariate analysis has shown no statistically significant difference between groups in all studied features, indeed both groups were similar in history, symptoms, biometric and biological measurements (Table 4).

**Table 4:** Description and between-arms comparison of the clinical features

**Variables**	Total	**Control group**	**Spirometry group**	***p*-value**
**Anamneses**				
History of cardiovascular disease: n (%)	83 (17.4)	44 (18.0)	39 (16.7)	0.725
History of pulmonary disease: n (%)	39 (08.1)	27 (11.0)	17 (07.3)	0.163
History of psychiatric disease: n (%)	13 (02.7)	8 (03.3)	5 (02.1)	0.452
History of diabetes: n (%)	88 (18.2)	48 (19.6)	42 (18.0)	0.662
Number of comorbidities: n (%)				
0	298 (62.3)	129 (52,7)	169 (72.5)	0.093
1	140 (29.3)	86 (35.1)	54 (23.2)	
2	38 (07.9)	29 (11.8)	9 (3.9)	
3	02 (0.4)	1 (0.4)	1 (0.4)	
Symptom, dyspnea: n (%)	295 (65.7)	152 (67.9)	143 (63.6)	0.337
Symptom, couth: n (%)	276 (61.6)	150 (61.7)	135 (57.4)	0.340
Symptom, chest pain: n (%)	197 (44.1)	107 (48.2)	90 (40.0)	0.081
Symptom, epigastralgia: n (%)	182 (40.6)	95 (42.6)	87 (38.7)	0.397
Symptom, dental damage: n (%)	332 (67.3)	173 (70.6)	159 (67.7)	0.484
Other symptoms: n (%)	29 (06.5)	12 (05.4)	17 (07.6)	0.356
**Clinical examination**				
Weight: mean ± SD	77.56 ± 17.08	76.22 ± 15.91	78,84 ± 18.08	0.126
Height: mean ± SD	174.52 ± 8.87	174.01 ± 07.87	174,99 ± 9.68	0.269
BMI: mean ± SD	25.54 ± 4.79	25.26 ± 04.70	25,79 ± 4.86	0.291
Systolic Blood Pressure (SBP): mean ± SD	124.81 ± 18.14	121.81 ± 19.52	121,72 ± 16.69	0.957
Diastolic Blood Pressure (DBP): mean ± SD	75.14 ± 13.27	75.50 ± 10.16	76,97 ± 10.41	0.198
Capillary glycemia (Dextro): mean ± SD	1.16 ± 0.45	1.21 ± 0.52	1,13 ± 0.39	0.137
CO-Oxymetry: mean ± SD	11.07 ± 6.750	10.63 ± 6.53	11,57 ± 6.98	0.159

### Quitting motivation

The quantification of the motivation toward cessation among the two groups of participants at baseline is important to detect a possible bias. We included in our questionnaire a variety of subjective and objective variables to quantify patient perception on the importance of quitting and his confidence to undergo the procedure successfully. In addition, we included variables in relation with patient history of previous attempts that was frequently suggested to be of value. We also classified patient intention in accordance with Proshaska transtheoretical model. Therefore, equivalent distribution between study arms was verified to prevent a probable bias. No statistically significant difference (*p*<0.05) was seen while comparing quitting motivation perception. Hence both groups can be considered statistically similar in motivational aspects at baseline (Table 5).

**Table 5:** Between arms comparison of the motivational aspects

Characteristics	Control group	Spirometry Group	*p*-value
Importance of quitting (score/10): mean ± SD	9.14 ± 1.89	9.37 ± 1.34	0.161
Quitting confidence (score/10): mean ± SD)	7.14 ± 2.88	7.38 ± 2.65	0.269
Longest previous cessation period: n (%)			
None	35 (16.1)	29 (14.8)	0.350
Less than 6 months	149 (68.7)	125 (63.8)	
Between 6 and 12 months	9 (04.1)	15 (07.7)	
More than 12 months	24 (11.1)	27 (13.8)	
Delay to the last cessation attempt: n (%)			
None	35 (16.3)	28 (14.4)	0.069
Less than 6 months	21 (09.8)	46 (23.7)	
Between 6 and 12 months	30 (14.0)	24 (12.4)	
More than 12 months	129 (60.0)	96 (49.5	
Main quitting argument: n (%)			
Health	207 (93.7)	207 (91.6)	0.781
Family	2 (0.9)	4 (1.8)	
Money	4 (1.8)	4 (1.8)	
Others	8 (3.6)	11 (4.9)	
Main quitting concerns: n (%)			
None	97 (43.7)	75 (36.4)	0.530
Stress	91 (41.0)	74 (35.9)	
Obesity	6 (2.7)	7 (3.4)	
Onset of a disease	20 (9.0)	33 (16.0)	
Other	8 (3.6)	16 (7.8)	
Prochaska: n (%)			
** Contemplation**	64 (29.1)	61 (29.2)	0.878
** Preparation**	45 (20.5)	49 (23.4)	
** Action**	101 (45.9)	85 (40.7)	
** Maintenance**	1 (0.5)	0 (0.0)	
** Relapse**	9 (4.1)	14 (06.7)	

### Follow-up and outcome

During follow up, it was important to precise if patient had received a complete and free pharmaceutical intervention using Nicopatch® and to verify its group similarity. Indeed, groups were similar in receiving free treatment with 70.3% of availability in control group and 71.9% in spirometry group with no statistically significant difference. Number of visits per rotation or otherwise the duration of clinical follow-up was significantly higher within intervention group (3.01 weeks for control group vs 5.74 for intervention group; *p*=0.00) witnessing better adherence to the program.

The smoking cessation rate at 6 months was significantly higher in intervention group (48.0% vs. 33.1% in control group; *P*=0.002). At 1 year endpoint, cessation rates dropped in both arms, but the proportion of patients who remained abstinent was significantly higher in intervention group with 25.5% abstinent rate (*n*=56) versus 16.5% in control group (*n*=39).

This difference was safely significant with *p*-value at 0.019 and expressed as a relative risk RR=0.89 (IC95%=0.81 to 0.96). The number needed to treat NNT=11.19 with 95% CI ranging from 6 to 66. Otherwise, considerable reduction was observed in both groups within nonquitters with no statistical significant difference (*p*= 0.349) (Table 6).

**Table 6:** Follow up and outcomes: between arms comparison of 6th month and 12th month cessation rates

Outcome variable	Control group	Spirometry group	*p*-value
Treatment availability: n (%)	70.3%	71.9%	0.757
Number of visits: n (%)	3.01	5,74	0.000
Six months—cessation rate n (%: IC95%)	78 (33.1:27.1–39.1)	106 (48.0: 41.4–54.6)	0.002
One year—cessation rate n (%: IC95%)	39 (16.5: 11.7–21.2)	56 (25.5: 19.7–31.3)	0.019
Smoking reduction (cigarettes / day) mean	10.05	12.22	0.349

Since the baseline characteristics intervention and control group showed almost no difference in major aspects, we can, within the limits of the study, conclude to a positive effect of the intervention on cessation rate.

## Discussion

### Spirometry for smoking cessation

The global adoption of tobacco control program has led to the emergence of many cessation intervention strategies. Pharmacologic interventions and cognitive-behavioral therapies are proven cessation methods [5, 16]. However, those methods may be helpful for people already motivated to quit but, in most cases, smoking remains a difficult habit to break for people with low motivation and among special populations [12].

Motivation is where the biggest challenge of smoking cessation prevails, therefore medical institutions and teams developed a variety of tools to enhance patient motivation toward cessation mainly through a presentation of the adverse effects of smoking versus the benefits if quitting and assistance during the quitting procedure [4].

This study was conducted mainly to answer the question: does a pulmonary functional test enhance the motivation to quit smoking and consequently increase cessation rates. Results showed that telling smokers the results of their spirometry and their lung age significantly improves the likelihood of their quitting smoking with an increase of cessation rates by 15% in 6^th^ month-point-prevalence and by 9% in 12^th^ month-point-prevalence.

Literature was relatively rich but inconclusive. For a more in-depth reading of our results, we extracted similar RCTs from the two principal systemic review on the subject [6, 7] and from other sources (mainly *PubMed* and *Google Scholar*). Selected studies were published from 1990 to 2017, three more protocols of ongoing trials were available online [17–19]. Selection was based on the criteria of spirometry being independently the subject of the study, and not used as a part of usual care in both arms but when it was performed and communicated as a motivator for the intervention group. Samples size was variable from 33 to 294 per arm, in contrast with our study where 236 individuals reached endpoint in control group and 220 in spirometry group.

Cessation rates at endpoint was very variable in the literature ranging from 0% to 78.6%. This wide range of difference can be explained by variability of the standard customized intervention for example usual care in Kaminsky et al. trial is a one-minute minimal cessation advice (cessation rate 24%) [20]. While in Takagi et al., the Japanese standard cessation program was used as usual care which is a rotation of 5 visit with detailed smoking cessation advice, physical examination an behavioral therapy (cessation rate 69%) [21]. Sample size can also be a decisive factor, along with the specifications of each population, in fact we expect a big variability between population from different countries and backgrounds. Therefore, appreciation of literature results was not on the absolute rates of cessation but on the added value of spirometry intervention regardless of correspondent local reference setting.

Hence, results was classed as follow: 1) a positive and significant impact of spirometry on cessation rate 2) a positive impact of spirometry on cessation rate but with no statistical significance 3) negative or no impact of the spirometry on cessation rate. Based on this classification, observed trials can be read as follow: two studies concluded to a positive effect of spirometry intervention on cessation rates [22, 23]. Six trials concluded to positive effect of spirometry on cessation rates but with not enough statistical power [20, 21, 24–27]. Two studies concluded to the absence of any effect of spirometry on smoking cessation rates [28, 29].

Those results confirmed our initial announcement of lack of clear evidence on the subject and leading to undergo this trial. Our outcomes were in favor of the efficacy of providing spirometry results as an encouragement for smoking cessation, yet the balance sheet is still inconclusive and the realization of more powerful RCTs on larger scale and metanalysis is highly recommended to clarify the vision on this subject.

In addition, providing patients with their spirometry results was related as an independent factor to an improve in dropout rates of smoking cessation programs, in our study duration of follow up was 3.01 week in control group vs 5.74 week at mean in spirometry intervention group. Comparable results were found in a review by Deane et al [30].

Another aspect to be discussed is the preference of a spirometric lung age announcement over a standard spirometry alone. Regarding that we provided both communications to patients in the intervention arm of our trial, this comparison was not possible in our case. Yet, a trial conducted by Parkes et al concluded for the superiority of spirometry results verbally delivered in the form of ‘lung age’ with a graphic display over a limited spirometry feedback (smoking cessation rate: 6.4% vs. 13.6% ; *p*=0.005) [31].

In this RCT, we relayed on patient self-reported cessation status and no biochemical validation such as expired carbon monoxide (CO) that can be done to confirm an authentic abstinence. Then, we resorted to phone survey for the endpoint evaluation. Most studies showed that self-reports of smoking status were accurate [32–34].

Besides, randomization was satisfying, sample size was acceptable comparing to similar studies reported in literature and loss rate was minimal (8.8%).

## Conclusion

To conclude, our study was an additional evidence for spirometry and lung age announcement as motivators for cessation. This study is considered eligible for the requirements of the main systemic reviews conducted on the subject, where a considerable methodological heterogeneity between studies was noted. Moreover, there is now stable evidence that factors like history of cessation and psycho-mental health are predictors of the success or failure of a smoking cessation attempt. Based on these findings we recommend the incorporation of lung health biomarking and communication in a multimodal approach including pharmacological assistance toward higher efficacy of our cessation program. Providing SCC and general practitioners’ offices by spirometry may be very beneficial for patients. It may increase motivation among smokers seeking cessation, change attitudes towards smoking cessation among non-motivated smokers, and also screen COPD for this at risk population [35, 36]. It is also advisable to restructure the clinic medical records toward a risk stratification model to predict and distinguish patient with higher risk of withdrawal and customize their rotations in order to enhance the likelihood of their success.

Finally, despite the development of several cessation strategies, smoking cessation is still a challenging procedure with a high risk of relapse. This requires apart from all that was previously discussed, the motivation of the medical corps to seek for and deploy every possible tool that can help in the fight against this global health scourge.

## Data Availability

The datasets generated and/or analyzed during the current study may be publicly available from the corresponding author after elimination of identifying information.

## Abbreviations

WHO: World Health Organization
RCT: Randomized Controlled Trial
SCC: Smoking Cessation Clinic
RR: Relative Risk
ARR: Absolute Risk Reduction
NNT: Number Needed to Treat
